# Research progress of plant medicine and Chinese herbal compounds in the treatment of rheumatoid arthritis combined with osteoporosis

**DOI:** 10.3389/fmed.2023.1288591

**Published:** 2024-01-11

**Authors:** Zhuoxu Gu, Guanghui Zhou, Xianquan Zhang, Guihong Liang, Xiao Xiao, Yaoxing Dou

**Affiliations:** ^1^State Key Laboratory of Traditional Chinese Medicine Syndrome/The Second Clinical College of Guangzhou University of Chinese Medicine, Guangzhou, China; ^2^The Second Affiliated Hospital of Guangzhou University of Chinese Medicine, Guangzhou, China; ^3^The Research Team on Bone and Joint Degeneration and Injury of Guangdong Provincial Academy of Chinese Medical Sciences, Guangzhou, China

**Keywords:** rheumatoid arthritis, osteoporosis, bone metabolism, traditional Chinese medicine, geriatric medicine, plant-based natural products

## Abstract

Rheumatoid arthritis (RA) is a chronic systemic autoimmune disease. The clinical manifestations of various joint pain and bone destruction are common. RA has a high disability rate and is closely related to local and systemic osteoporosis (OP). RA can occur at any age, however, its incidence increases with age. Most patients are 40 to 50 years old with an incidence among women approximately 3 to 5 times more than among men. Osteoporosis is a kind of metabolic bone disease characterized by bone mass and bone microstructure damage and is one of the common complications of RA. Currently, in the clinic, more patients develop RA with OP symptoms. Therefore, both OP and RA-related factors should be considered in the OP treatment of RA. Currently, there is more and more research on RA combined with OP drugs, including basic drugs, bone resorption inhibitors, bone formation promoters, and anti-rheumatic drugs to improve the condition. The high cost or limited efficacy of certain Western drugs, coupled with their potential for adverse reactions during treatment highlight the pressing need for novel pharmaceuticals in clinical practice. In recent years, traditional Chinese medicine (TCM) can improve the bone formation and bone resorption indexes of patients with RA, regulate the balance of osteoclasts and osteoblasts, and regulate the immune inflammatory response, so as to treat RA combined with OP. This article discusses the advancements in single Chinese medicine and Chinese medicine combination treatments for RA complicated with OP, focusing on the mechanism of action and syndrome differentiation and classification, to offer new ideas for future clinical prevention and treatment.

## Introduction

Rheumatoid arthritis (RA) is a chronic autoimmune disease that leads to synovial cell proliferation and pannus formation in newborns. The occurrence, development, and production of RA are closely correlated with inflammatory cell infiltration and capillary involvement in the joints. The main clinical manifestations include pain, swelling, deformity, and functional decline. In addition to joint involvement, trabecular bone destruction, reduction in bone mass, and increased bone fragility are secondary effects. Studies have shown that patients with rheumatoid experience increased bone resorption and a higher incidence of osteoporosis (OP) (1). A Korean epidemiological study revealed that nearly 50% of postmenopausal women with RA had osteoporosis. In contrast, the prevalence of osteoporosis among Korean adults over 50 years old is 35.5% for women and 7.5% for men (2, 3). Currently, the pathogenesis of RA-induced OP remains poorly studied. However, it primarily involves factors such as receptor activator of nuclear factor κB ligand (RANKL), receptor activator of nuclear factor κB (RANK), the osteoprotegerin signal transduction pathway system, inflammatory mediators, and pharmacological agents such as glucocorticoids—all of which can contribute to the pathogenesis of OP (4). OP, the most common complication of RA, significantly impacts the treatment effectiveness, prognosis, and quality of life for patients (5). Currently, there are various drugs available with their own advantages and disadvantages, particularly concerning adverse reactions in the gastrointestinal tract that hinder patient acceptance and lead to poor compliance. Traditional Chinese medicine (TCM) monomers and their compound medicines have gradually proved to be significantly effective in treating RA combined with OP. The advantage lies in treating patients’ symptoms and improving their constitution based on clinical symptoms and TCM constitutional defects. The research on the pathogenesis of RA leading to OP has increased, but there is insufficient research in the field of TCM to explain how TCM treats RA combined with OP (6). Therefore, it is necessary to comprehensively study recent research in traditional Chinese medicine prevention and treatment of RA combined with OP. This article aims to accumulate clinical experience and provide new treatment ideas.

## Understanding of RA combined with OP in TCM

Modern medical theory posits that the kidneys primarily assume responsibility for metabolite excretion and body fluid balance regulation. This concept is more specific and limited, diverging significantly from the TCM interpretation of the “kidney.” TCM scholars perceive the kidney as a functional complex encompassing aspects of renal function, endocrine regulation, nervous system involvement, and other systems in modern medicine. Notably relevant to rheumatoid arthritis combined with osteoporosis is the belief in TCM that the kidney harbors essence while governing bone health and marrow vitality. Chinese medicine scholars believe that kidney deficiency is the key to the occurrence of rheumatoid arthritis combined with osteoporosis. According to the pathogenesis and clinical symptoms of RA combined with OP, Chinese medicine classifies it into the category of “bone Bi” and “Bi syndrome.” “Medical Jing Yi” explained that when the kidney qi is abundant, it nourishes the kidney essence, promotes active marrow generation, strengthens and densifies the bones, and allows for free movement of limb joints. On the contrary, when there is a deficiency in kidney essence, the bone marrow lacks a source of biochemicals and insufficient marrow essence cannot support bone health. As a result, weak bone tendons and bone destruction may occur. Qing Dynasty physician Wang Qingren put forward the theory of “stasis causing Bi,” and Lin Peiqin that “stagnation for a long time must be blood stasis.” Therefore, the invasion of external evil causes the meridians to be blocked and a deficiency of Qi and blood, and blood stasis is formed for a long time. Now medical science has found that RA patients have abnormal hemorheology and blood circulation disorders, which can cause blood stasis and venous stasis, aggravate microcirculation disorders around RA joints, and lead to abnormal calcium absorption, which damages the joint synovium and bone tissue. This can eventually lead to secondary osteoporosis (7). As mentioned previously, there are many mechanisms of RA causing OP, and the clear causes need to be studied. Studies have found that rheumatoid arthritis patients exhibit varying degrees of bone mineral density loss, which is associated with disease activity, duration, inflammatory markers, and other factors. Dual-energy X-ray absorptiometry results indicate decreased bone mineral content in the femur and lumbar spine of rheumatoid arthritis patients (8). Assessing fracture risk in patients with rheumatoid arthritis and osteoporosis is crucial. Studies have demonstrated that a comprehensive assessment method combining clinical indicators, bone mineral density detection, and TCM syndrome characteristics improves the accuracy of predicting fractures. Early diagnosis and treatment of underlying conditions along with lifestyle changes can help increase bone mineral density to prevent fractures (9). Studies have found that TCM syndromes in RA patients with osteoporosis mainly include kidney deficiency, liver depression, and spleen deficiency. In terms of TCM treatment, tonifying the kidneys, soothing the liver, and invigorating the spleen can effectively improve symptoms (10).

## Treatment of RA with OP by TCM monomers

**Figure 1:**
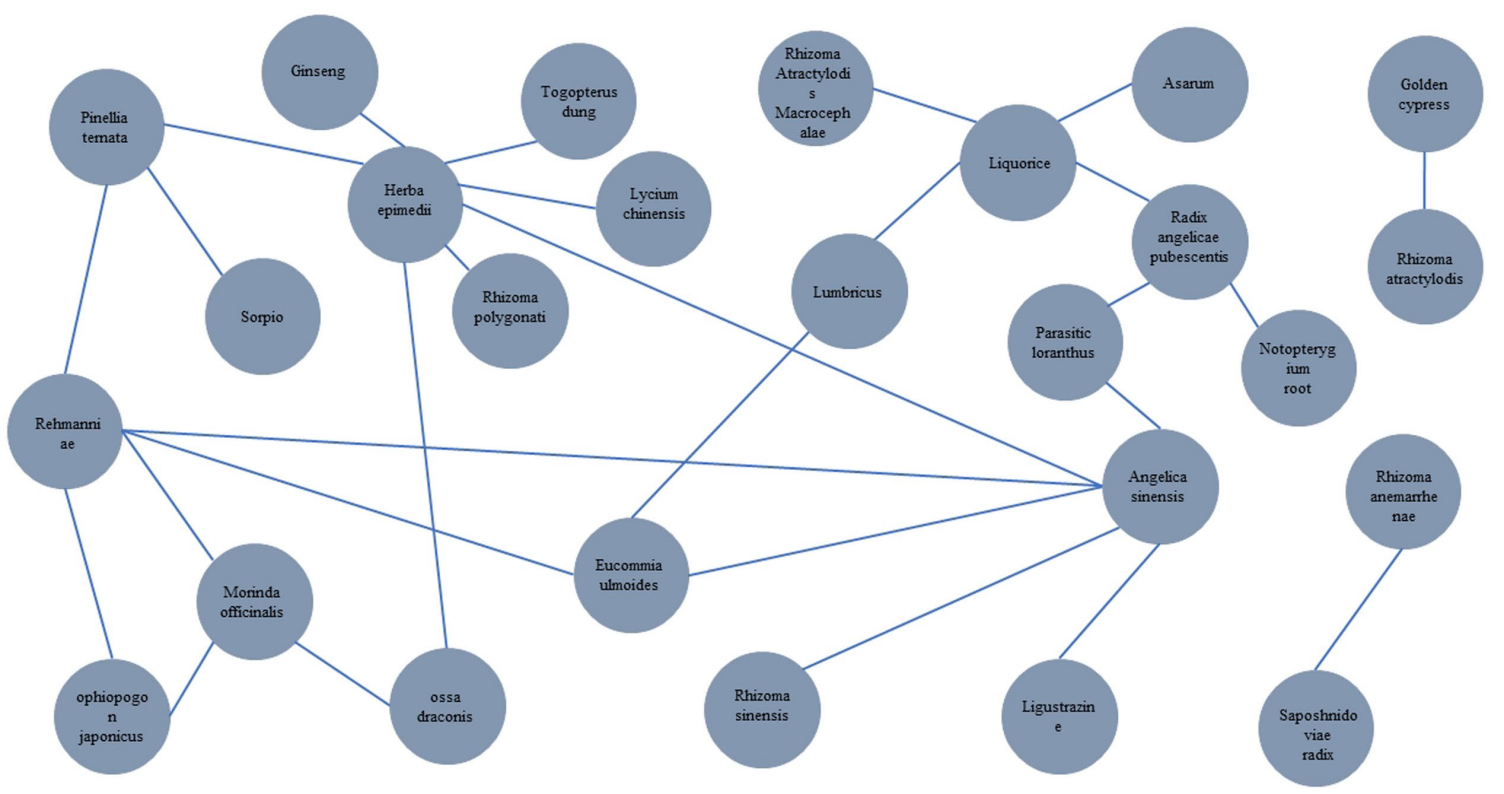
Visualization of the interaction network of drugs for the treatment of RA and OP.

When herbal medicine is used to treat RA and OP, there is an overlap in the traditional Chinese medicines commonly employed for both diseases. By conducting an analysis of the TCM prescription patterns for RA and OP, Zhong Liyan discovered that in the treatment of RA, there is a strong correlation with the administration of white mustard seed, honeysuckle, Lonicerae japonicum, Tripterygium wilfordii, ground beetle, turmeric, Kadsura pepper stem, Rhizoma Dioscoreae Nipponicae, and Paniculate Swallowwort (11). Similarly, in the treatment of OP, Moutan PI, Amphorae fructus, Dipsacus, Pinnellia pinellia, Morinda officinalis, and Ligustri lucidum were found to have significant correlations. The advantage of herbal medicine in the treatment of RA combined with OP is that the mechanism of action of traditional Chinese medicine in the treatment of osteoporosis is achieved through systemic, multi-link, and multi-pathway regulation. The Chinese medicine monomers for the treatment of RA and OP with their interactions are shown in Figure 1 and Table 1.

**Table 1:** Chinese herbs used to treat RA and OP.

Herbs	Main active ingredient	Clinical effect	Reference
Tripterygium Hypoglaucum (Levl.) Hutch	Triptolide, triptolide triol, triptolide triterpenoid quinone A, and triptolide triterpenoids	Immunosuppressive, anti-inflammatory, osteoclastogenesis-inhibiting	(12)
Radix angelicae pubescentis	RA: Methoxyparsley and dihydroparsley OP: Osthole Cnidium lactone	RA: Anti-inflammation, anti-oxidation, and eliminate wind and dampness OP: Inhibition of osteoclast bone absorption and bone metabolism	(5) (6)
Rhizoma Drynariae	Naringin and Drynaria total flavonoids	RA: Inhibition of inflammatory reaction OP: Anti-osteoporosis and renal protection	(13) (14)
Radix Cyathulae (achyranthes bidentata)	Ecdysterone (ECR)	Anti-apoptotic and anti-inflammatory	(15)
*Salvia miltiorrhiza* Bunge (Tanshinone)	Salvianolic acid B and Tanshinone VI	RA: Anti-inflammatory and anti-oxidation OP: Increases bone mineral density and promotes bone formation	(16) (17)
Angelica sinensis (Oliv.) Diels	Angelica including volatile oil, organic acids, amino acids, and coumarin.	Analgesic, anti-inflammatory, and anti-osteoporosis	(18)
Radix paeoniae alba	Albiflorin std., triterpenoid, and flavonoid	Analgesic, anti-inflammatory, antidepressant, and regulates immune system	(19)
Red peony root	Albiflorin std., triterpenoid, and flavonoid	Anti-inflammatory, anti-oxidation, nerve protection, and improves microcirculation	(20)

### Tripterygium hypoglaucum (Levl.) Hutch

The monomeric extract of Tripterygium hypoglaucum (Levl.) Hutch, known in traditional Chinese medicine, is derived from the root of this plant, which is a unique variety belonging to the genus Tripterygium in the Epimonaceae family. It has an effect on the three channels of the liver, spleen, and kidney. It has a bitter, punic, and mild taste, and has the functions of dispelling wind and removing dampness, promoting blood circulation and stopping bleeding, soothing tendons and bone healing, etc. Its main active ingredients include diterpenoids such as triptolide, triptolide triol, triptolide triterpenoid quinone A, and triptolide triterpenoids (21). According to the introduction, the basic pathological change of RA is the chronic inflammation of the synovial membrane of the joint, which leads to pannus formation, invasion of articular cartilage and subchondral bone, bone resorption, bone destruction and osteofibrosis, and finally joint malformation and loss of function. On the one hand, Tripterygium hypoglaucum Hutch can be used for therapeutic purposes for RA combined with OP by regulating bone metabolism. Mo Danya et al. (22) found by observing joint X-ray films of experimental mice that tripterygium, the active ingredient in Malus Kunmingshan, can inhibit synovium hyperplasia, antagonize cartilage destruction, improve bone density, and prevent osteoporosis. In addition, triptolide can significantly reduce the levels of vascular endothelial growth factor (VEGF) in synovial tissue and IL-6 in synovial fluid of joints, as well as the formation of new vessels in synovial tissue of rats, inhibit the proliferation of synovial cells, and show protective effects on synovial tissue and cartilage tissue (23). Liu et al. (24) found that triptolide could inhibit osteoclast formation by reducing the expression of nuclear transcription factors and osteopG in the joint cavity at the mRNA and protein levels, confirming that triptolide could inhibit osteoclast formation. On the other hand, in the prognosis of RA combined with OP, due to long-term and heavy use of anti-rheumatic drugs, adverse reactions may cause kidney damage. Studies (25) have pointed out that renal dysfunction with no obvious clinical manifestations is more common in RA patients, kidney involvement has become an important factor affecting the prognosis of RA, and renal failure is also a common cause of death for RA. In addition, traditional Chinese medicine states that “the kidney governs the bone,” so in the prognosis and treatment of RA combined with OP, taking into account the protection of the kidney is also the key to preventing and treating the symptoms of osteoporosis. Zeng Hongbing et al. (26) found that Tripterygium hypoglaucum Hutch may reduce proteinuria and delay kidney injury by reducing the expression of transforming growth factor, inhibiting the proliferation of mesangial mesangium. Wu Xiabo et al. (27) found that the therapeutic effect of Malus Kunmingshan on nephrotoxic nephritis in rats was achieved by reducing urinary protein content, serum urea nitrogen, triglyceride, and total cholesterol, increasing serum albumin and total protein, improving renal function and pathological changes of glomerulus.

### Radix angelicae pubescentis and other monomer TCMs with a similar mechanism

Radix Angelicae Pubescentis has the effect of dispelling wind dehumidification, relieve pain and dredging channel blockage. Radix Angelicae Pubescentis was first published in the “Shen Nong Bencaojing,” which said that “it [is] mainly used in the treatment of wind-cold damp pathogen and incised wound knife injurious to health. And treat all kinds of orthopedic diseases caused by wind evil entering the channels.” Methoxyparsley and dihydroparsley are two of the most active ingredients in Lonosol. Studies have shown (28) that methoxyparsley is the main component of the antitumor, anti-angiogenesis, and anti-proliferation properties of doxine. The anti-inflammatory activity of dihydroparsley has been confirmed, but its mechanism is still unclear. In recent years, it has been confirmed that inflammatory factors, as inflammatory mediators, on the one hand, led to the occurrence of the primary disease of RA, and on the other hand, destroy the normal bone metabolic balance of the body and affect bone loss (29). Chao et al. (30) studied dihydroparvyl angelic acid and found that it inhibits the LPS-mediated inflammatory response mainly by inhibiting the activation of nucleotide-binding oligomerization domain 1 (NOD1) /NF-κB. Naringin, the active ingredient of the osteocalcin supplement, can promote the differentiation and proliferation of bone marrow stromal cells, increase the expression of osteocalcin, and effectively reverse the process of osteoporosis. Ang et al. (31) suggested that naringin inhibited the activation of NF-κB by inhibiting the degradation of IκB-α mediated by RANKL, and inhibited osteoclast generation and bone resorption by interfering with the RANKL-mediated NF-κB and extracellular signal-regulated kinase ERK signaling pathways. Achyranthes root extract can improve the biomechanical quality of bone and trabecular structure (32). Ecdysterone (ECR) is the main component of achyranthes and has been used for the prevention and treatment of osteoarthritis. Zhang et al. (15) showed that ECR plays an anti-apoptotic and anti-inflammatory role in rat chondrocytes induced by interleukin-1β, which may be related to the NF-κB signaling pathway. It has also been demonstrated that salvianolic acid B prevents bone loss in glucocorticoid-treated rats by stimulating osteogenesis and bone marrow angiogenesis, and inhibiting fat formation (33). Nicolin et al. (34) demonstrated that tanshinone VI inhibits osteoclast differentiation by inhibiting the expression of RANKL and NF-κB. In summary, dysregulation of the NF-κB signaling pathway may be the basis of the pathogenesis of RA complicated with OP. The aforementioned herbs can exert a favorable therapeutic impact on RA complicated with OP by modulating the NF-κB signaling pathway. In conclusion, the pathogenesis of RA complicated with OP is related to the interaction between signal transduction pathways and inflammatory factors and is closely related to vitamin D level and physical exercise. The effect of RA treatment drugs on OP should not be underestimated.

### Angelica sinensis

Angelica sinensis, which is medicinal sweet, pungent, humoral, and returns to the liver, heart, and spleen channel, has the effect of tonifying blood and promoting blood circulation, regulating the menstrual flow, and relieving pain, moistening the bowels. The main chemical components of Angelica include volatile oil, organic acids, amino acids, and coumarin, which have pharmacological effects such as analgesic, anti-inflammatory, anti-osteoporosis, anti-platelet aggregation, anti-anemia, and protection against cardiovascular and cerebrovascular diseases (18). The inflammation in RA is one of the causes of OP. Studies by Ma et al. (35) have shown that Artemisonolactone, an extract of Angelica sinensis, has obvious anti-inflammatory effects on ovariectomized rats with osteoporosis. Ferulic acid, an effective component of angelica, can reduce the expression of IL-1β, TNF-α, matrix metalloproteinase-1, and matrix metalloproteinase-13 in chondrocytes induced by hydrogen peroxide, thus playing a protective role in articular cartilage (36).

### Radix paeoniae alba and red peony root

Paeoniae alba, which is medicinal bitter, acidic, slightly cold, and returns to the liver and spleen meridian, has the effect of nourishing blood and regulating the meridian, collecting Yin and stopping perspiration, soothing the liver, and relieving liver Yang. Modern pharmacological studies have shown that Paeony has anti-inflammatory, analgesic, immune regulation, liver protection, anti-depression, and other pharmacological effects, and has been widely used in the clinical treatment of RA, chronic hepatitis B, cancer pain, and other kinds of pain (19). Studies have shown (37) that Paeony can reduce inflammation in RA and delay the bone destruction and progression of RA by reducing the levels of IL-1, IL-1β, IL-17, and TNF-α, inhibiting the activation of NF-κB and the proliferation of synovium and regulating the levels of IL-2 in both ways and through other mechanisms.

Red peony root, which is bitter, slightly cold, and affects the liver channel, has the effect of clearing heat and cooling blood, dissipating blood stasis, and relieving pain. Modern pharmacological studies have shown that peony has various pharmacological effects such as protecting nerve cells and cardiomyocytes, anti-atherosclerosis, lowering pulmonary artery pressure, anti-thrombus, stabilizing microcirculation, hypoglycemic, anti-gastric ulcer, protecting the liver, anti-depression, antiviral, anti-inflammatory, anti-tumor, and anti-radiation. It has been widely used in the treatment of circulatory, nervous, blood, digestive, and other multi-system diseases (38). Qin Yanqin et al. (39) studied the effects of the effective components of Paeonia lactis on inflammatory factors in an A549/THP-1 cell co-culture inflammatory model, and found that it could reduce the levels of IL-1β, IL-6, IL-23, and MMP-9, thereby inhibiting the inflammatory response.

In conclusion, although there is insufficient evidence to strictly infer the use of Chinese medicine monomers in treating RA-OP, the above studies have demonstrated their potential efficacy in reducing inflammation and oxidative stress. Although many studies have shown the anti-inflammatory and anti-oxidative effects of TCM monomers, as well as their therapeutic potential for RA-OP in laboratory and animal experiments, further verification is needed for clinical application. To more fully evaluate the efficacy of TCM monomers in treating RA-OP, attention should be given to the following studies: (1) Large randomized double-blind controlled trials are the most reliable way to assess drug efficacy and safety. More such trials on using TCM monomers for treating RA-OP will help confirm their clinical efficacy. (2) Clinical case reports and retrospective analysis provide initial clinical data on using TCM monomers for treating RA-OP, although their reliability is relatively low. Paying attention to this literature helps one understand the application value of TCM monomers in actual clinical practice. (3) Research on the mechanism of drug action helps optimize treatment plans and predict possible side effects by gaining an in-depth understanding of how Chinese medicine monomers affect RA-OP.

## TCM prescription to treat RA combined with OP

**Figure 2:**
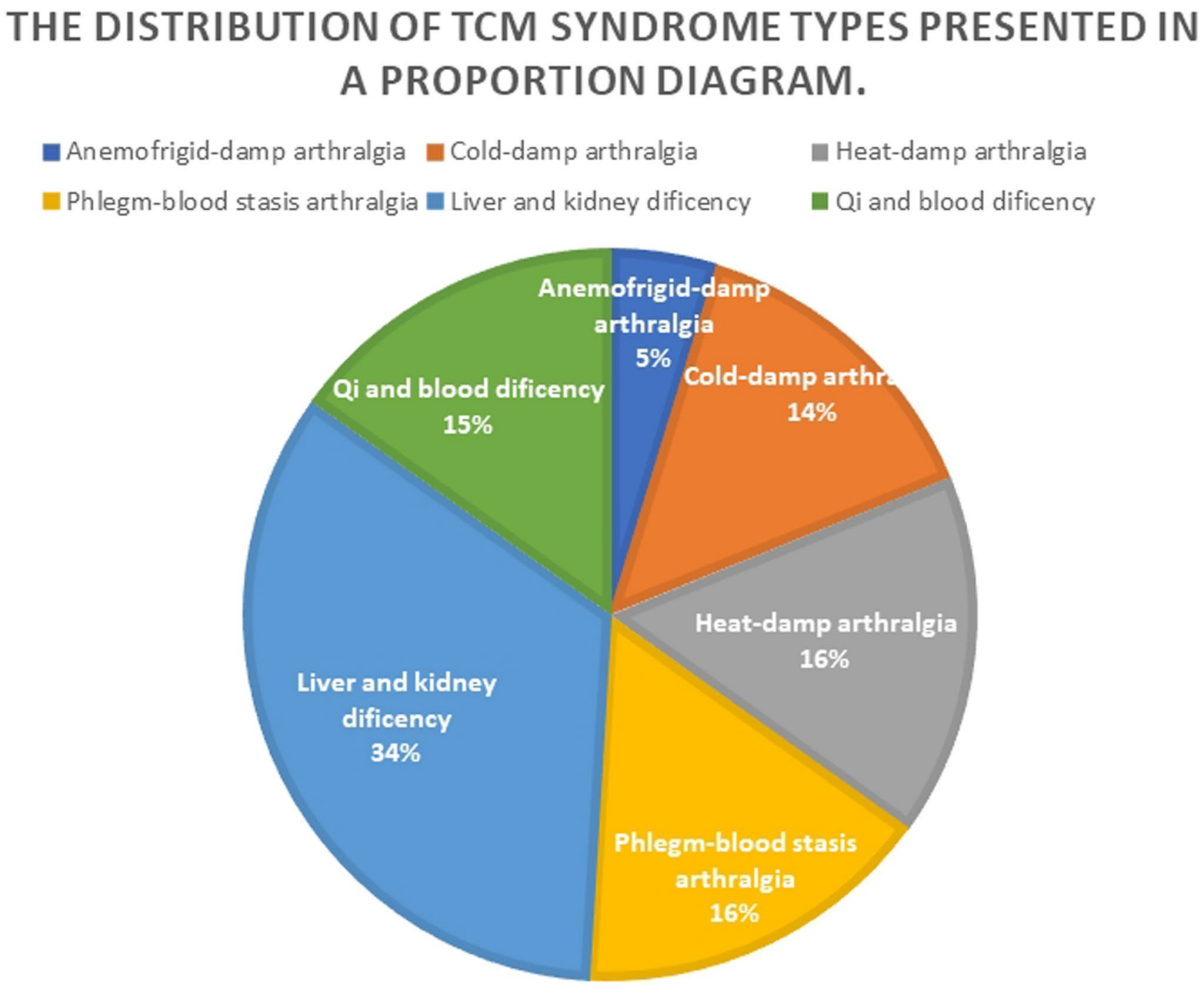
Proportion diagram of TCM syndrome types distribution.

In 2002, the Guiding Principles of Clinical Research on New Chinese Medicine (Trial Implementation) divided RA into five syndrome types: damp-heat obstruction, cold-dampness obstruction, kidney qi deficiency and cold, liver and kidney Yin deficiency, and blood stasis obstruction (40). Zeng Zhaoyang et al. (41) classified osteoporosis into four syndrome types: kidney Yang deficiency syndrome, liver and kidney Yin deficiency syndrome, spleen and kidney Yang deficiency syndrome, and blood stasis and qi stagnation syndrome. Zhang Hongyue (42) clinical study on traditional Chinese medicine syndromes found that among 161 RA patients with OP, wind-cold-dampness syndrome accounted for 4.96% (8 cases), cold-damp obstruction syndrome accounted for 14.90% (24 cases), damp-heat obstruction syndrome accounted for 16.77% (27 cases), phlegm and blood stasis obstruction syndrome accounted for 17.39% (28 cases), qi and blood deficiency syndrome accounted for 9.93% (16 cases), and liver and kidney deficiency was diagnosed in 36.02% of the cases (58 cases), as shown in Figure 2.

### TCM compound of tonifying kidney and dredging collaterals

Kidney deficiency is the key to the occurrence of rheumatoid arthritis combined with osteoporosis. Medical Jing Yi states that the kidney qi is sufficient; the kidney essence is abundant; the medullary generation is active; the bone is dense, firm, and powerful; and the limbs and joints move freely. On the contrary, the kidney essence is deficient, the bone marrow is biochemically deficient, and the medullary essence is insufficient, so the bones and tendons are weak and the bone is destroyed. Therefore, the treatment of arthralgia should take the specimen into account, dispelling the evil. Wang Fang (43) found that a Pei-Bushenyang Decoction supplemented with flavor could significantly relieve clinical symptoms in RA combined with OP patients; reduce the levels of ESR, CRP, and BALP; increase the indexes of 25-OH-D, osteocalcin, and blood calcium; and improve bone mineral density, with significant differences. On the basis of Western medicine treatment, Tian Jiexiang (44) added a representative prescription of a Bushentongluo Gutabaikang pill (antler cream, ejiao, bone crushing supplement, cinnamon, turtle plate, turtle shell, etc.) to treat the disease, and the patients’ clinical symptoms were alleviated and bone mineral density was significantly improved. According to many years of clinical experience, Professor Pang Xuefeng (45) summarized the empirical prescription for the treatment of RA in the active stage: a Hanbikang Decoction, which is composed of kidney toning drugs such as epimedium and Guji and anti-rheumatic drugs, can significantly improve the clinical symptoms and signs of patients with RA combined with OP, and the improvement of bone mineral density is better than that of Western medicine alone. In addition, the animal experiments with the Hanbikang Decoction have also proved that it can reduce the expression of RANKL and RANK in bone tissue and increase the expression of OPG by affecting the RANKL/RANK/OPG signaling pathway, so as to achieve the effect of anti-RA bone erosion and can be used to treat RA combined with OP. To sum up, traditional Chinese medicine kidney prescriptions for patients with RA and OP, alongside kidney anti-rheumatism medicine, can effectively improve the clinical curative effect, low bone mineral density values, morning stiffness, joint pain, swelling, and wait for a symptom, and is worthwhile for clinical use.

### TCM compound for liver and kidney deficiency

Professor Fan Yongsheng believed that Yin deficiency in the liver and kidneys can be seen in the early, middle, and late stages of RA (46), and advocated that nourishing the liver and kidneys should run through the whole process of RA. Clinical treatment of RA combined with OP syndrome of liver and kidney deficiency has achieved satisfactory clinical efficacy. According to Wang Xiaoqing (47), Zhiyin Yigu prescription (Rehmannia rehmannii, Mulberry parasitic, Achyrano rhizoma, yellow juniper, Euperia ulmoides, stucca, etc.) has the functions of nourishing liver and kidney, dispelling wind and dampness, clearing heat deficiency, and inhibiting osteoclast activity by increasing the content of OPG and RANKL in serum, thereby reducing bone loss and controlling the development of disease. It has a definite effect on RA combined with OP patients with liver and kidney deficiency syndrome. In addition, the core prescription for the treatment of RA combined with OP is Duhuo parasitic decoction, which has been proved by modern pharmacology to inhibit platelet aggregation, inhibit thrombus, and facilitate the blood circulation of joints. The ethanol extract in mulberry parasitic can dilate blood vessels and has the effect of reducing blood pressure. Rhizoma Drynariae can promote the absorption and utilization of calcium in bones, increase the levels of calcium and phosphorus in blood, improve chondrocytes, delay cell degeneration, reduce the incidence of bone and joint lesions, and play a sedative and analgesic role (48). A Liuwei Dihuang pill with cooked Rehmannia nourishes the liver and kidneys, benefits the essence, and fills the marrow. According to Compendium of Materia Medica, it can “fill bone marrow, grow muscle and produce essence blood.” Modern pharmacological studies (49) show that a Liuwei Dihuang pill can inhibit Th2-mediated immune inflammation, inhibit overexpression of Th2, regulate the balance of Th1 and Th2, and enable patients to achieve a balanced immune function. Zhou Jinsheng (50) observed the treatment of 88 patients with rheumatoid disease and found that the combined treatment with Liuwei Rehmannia could reduce the blood sedimentation rate index and reduce the inflammatory response of patients, which was helpful in improving clinical symptoms. Long Kuanbin et al. (51) used a Liuwei Dihuang pill to treat 60 patients with rheumatoid disease complicated with osteoporosis and the patients’ clinical symptoms were improved.

### TCM prescriptions for resolving phlegm, promoting blood circulation and pathways

Professor He Dongyi (52) argues that the pathological basis of RA combined with OP is based on the deficiency of kidney essence, the blockage of blood stasis as the standard, the congenital deficiency of kidney deficiency leads to the loss of the essence of the muscles, bones, and joints to replenish the blood, the warmth of the kidney Yang, and the evil of wind and cold dampness invading the human body, blocking the Qi and blood, making the operation of Qi and blood not smooth, the long-term phlegm and blood stasis interlock, the muscle and bone dystrophy, causing joint pain and deformity and eventually leading to osteoporosis. Li Xiaodan et al. (53) treated 76 patients with rheumatoid arthritis with a Dianteng Yimu decoction, and found that bone metabolism indexes were improved compared with before treatment, confirming that a Dianteng Yimu decoction can inhibit bone absorption in patients with rheumatoid arthritis after treatment, especially in promoting bone formation. Jiang Yi et al. (54) treated RA combined with OP patients with Bixie Qufengyin combined with methotrexate. After treatment, the joint function index, bone pain index, joint swelling index, joint tenderness index, and morning stiffness time of patients in the observation group were decreased compared with those before treatment and the control group, and the difference was statistically significant, indicating that Bixie Qufengyin combined with methotrexate achieved the clinical therapeutic purpose. Zhong Weijing et al. (55) discussed the mechanism of action of Shexiang Oolong pills (artificial musk, Radix aconitum, Radix aconitum, Radix scorpion, Radix black beans) from the perspective of cytokines. The Chinese medicine uses musk as its principal component, which can move the stagnation in the blood, promote blood circulation, and diffuse the nodules. Musk is also the most fragrant fragrance, which can penetrate the meridians and collars, so it can relieve pain. Studies have shown that Musk Oolong pills can increase the serum OPG level and reduce the level of RANKL in patients with RA, inhibit bone resorption, regulate bone metabolism, and then delay bone destruction and promote bone repair.

To conclude, TCM prescriptions can well improve RA combined with OP clinical symptoms. Chinese herbal formulas have the potential to significantly improve the treatment of RA combined with OP therapy, offering a wide range of opportunities for development in this field. The micro-therapeutic effects mentioned above are primarily mechanical, but some studies have demonstrated the efficacy of TCM in treating RA combined with OP. For instance, a randomized controlled trial involving 40 RA patients with OP revealed significant improvements in pain levels, joint inflammation symptoms, and quality of life after 24 weeks of TCM treatment (56). In another clinical study with 60 RA patients suffering from OP, those treated with traditional Chinese medicine experienced notable alleviation in symptoms such as joint pain, swelling, and stiffness after 24 weeks of treatment while also reducing the risk of fractures (57). Furthermore, a separate study involving 120 RA patients with OP found that traditional Chinese medicine led to a significant reduction in serum inflammatory factor levels (such as tumor necrosis factor-α and interleukin-6) after 12 weeks of treatment along with improved bone mineral density (58). Although not all of the studies mentioned above were randomized controlled trials (RCTs), they demonstrated TCM treatment’s efficacy in improving clinical symptoms and bone metabolism in RA patients with OP.

## Discussion

In recent years, with the deepening of research on rheumatoid arthritis, more and more attention has been paid to the situation of bone destruction and systemic bone loss caused by RA combined with OP. OP is a difficult condition and a key point in the clinical treatment of RA. TCM has accumulated a lot of practical experience in the treatment of RA combined with OP. It has also achieved ideal results, and has its unique advantages and characteristics in syndrome differentiation and multi-target multi-approach holistic treatment. At present, there is no expert consensus on TCM syndrome differentiation and treatment of RA combined with OP. Researchers have mostly carried out relevant studies based on the traditional Chinese medicine syndrome types of RA. Although the classification criteria of RA are not the same and there are few relevant studies, the existing results mostly show that patients with RA syndrome of liver and kidney insufficiency and phlegm and blood stasis are more serious in bone destruction and more likely to be complicated with OP. In terms of treatment, many methods such as supplementing the liver, spleen, and kidneys; eliminating phlegm; promoting blood circulation; and clearing collars are adopted. Western medicine also has certain advantages and limitations in the treatment of RA combined with OP. In terms of advantages, Western medicine has clear targets and drugs for the treatment of RA and OP, such as biological agents, anti-resorption drugs, and drugs that stimulate bone formation, and the curative effect is clear. In terms of limitations, the method of Western medicine in the treatment of RA and OP is relatively myopic, focusing on drug therapy, and some drugs have side effects, which may affect the quality of life of patients (59). In view of the above-mentioned, there are still many mechanisms that are not exact. For example, how botanic drugs interact with inflammatory factors IL-1, IL-17, IL-6, and IL-4 in the RANKL/RANK/OPG system, and what role each inflammatory factor plays in regulating bone metabolism. In addition, the specific mechanism of action of Chinese medicine prescriptions is still not completely clear. Furthermore, for RA combined with OP, the theoretical documentation of Chinese medicine is not complete, and there are few studies on syndrome differentiation and classification. Compared with Western medicine intervention methods, the use of TCM internal and external treatment intervention in clinical treatment still has limitations. Finally, at present, there are more and more animal experimental studies on osteoporosis by traditional Chinese medicine researchers, but most of them only focus on a single disease of osteoporosis, mainly in the field of postmenopausal osteoporosis (PMOP), and there are few studies on RA combined with OP. In addition, the use of plant medicine and TCM prescription for treatment is the “internal treatment” in the concept of TCM. In the aspect of “external treatment,” there is still a lack of existing research, but relevant clinical studies have shown that the “external treatment” of traditional Chinese medicine has a certain therapeutic effect on RA combined with OP. Wu Mingxia et al. (60) treated 18 female patients with OP with acupuncture, and the main acupoints were Dazhu, Dazhui, and Mingmen. The bone mineral density of the lumbar spine and femoral neck was increased after treatment at Xuanzhong (GB 3), Geshu (BL 23), and Zusanli (ST 36). Xiong Fangli et al. (61) collected the auricular points of the uterus, kidneys, endocrine, ovaries, and spleen. The needles were retained for 2 days, and the two ears were treated alternately. Thirty days of solid embedding was taken as a course of treatment. After 3 courses of treatment, the symptoms, signs, X-ray films, bone mineral density, and urine calcium of 60 middle-aged and elderly women with OP combined with fracture were improved by 95%. Therefore, we believe that the improvement of the existing treatment scheme combined with the external treatment of TCM may achieve twice the result with half the effort, with traditional exercises such as Tai chi, Wu qinxi, Ba duanjin, possibly being a measure to improve the quality of life in the late stage of RA combined with OP. Acupuncture and moxibustion can promote the relief of symptoms. Auricular intradermal needling is one of the non-drug treatment methods, which can stimulate acupoints such as the liver, kidneys, and spleen, to achieve the effect of invigorating the spleen and nourishing the liver and kidneys.

## Conclusion and Prospect

In summary, epidemiological survey data has confirmed the presence of osteoporosis secondary to RA, which has gained increasing attention from rheumatologists. Western medicine primarily employs combination therapy, administering traditional anti-osteoporosis drugs alongside RA treatment. With the rapid development of traditional Chinese medicine, research on RA combined with OP is progressing and showing remarkable clinical efficacy. Clinical practice has demonstrated that utilizing TCM syndrome differentiation and treatment, focusing on holistic therapy, effectively alleviates the disease with minimal side effects for improved patient quality of life. Extracting monomers from single TCM ingredients to produce medicines offers clear quantification and accurate testing in laboratories while enjoying high recognition and acceptance abroad. This enhances the competitiveness of TCM in international markets and represents a direction for successful internationalization. Simultaneously, there will be increased and in-depth research on the causes, development, differentiation of symptoms, and treatment of rheumatoid arthritis with osteoporosis (OP) in the future. Efforts will be made to reach an academic consensus as soon as possible to fully utilize the unique advantages of TCM in treating rheumatic diseases. Therefore, it is necessary to deepen research on its mechanism of action through improving scientific and rigorous experimental design, conducting large-scale observations, and utilizing big data for research purposes, as well as engaging in effective interdisciplinary collaboration that provides a solid theoretical foundation and detailed data support for clinical practice. Research on TCM treatment for RA combined with OP has broad application prospects; however, further discussion is needed regarding its pathogenesis which plays a key role in medical disease treatment. Additionally, we suggest focusing future efforts on multidirectional and multitargeted research into plant medicine mechanisms and treatment of osteoporosis and rheumatoid arthritis with TCM compounds at metabolomic, proteomic, and cellular molecular gene levels while addressing the aforementioned research limitations. In terms of clinical treatment approaches, we believe that strengthening health education about OP among RA patients should be prioritized. We should promote a healthy lifestyle and control all factors related to osteoporosis. Additionally, we should monitor bone metabolism indexes, BMD, and fracture risk assessment in RA patients with OP during diagnosis and treatment. It is important to advocate for RA patients to receive the lowest possible dose of glucocorticoids to reduce the risk of osteoporosis. In clinical practice, we need to consider drug efficacy, adverse reactions, and treatment costs based on each individual’s situation in order to promptly prevent and treat osteoporosis while relieving symptoms of RA, diminishing fracture risks, and bringing greater benefits to patients.

## Author contributions

ZG: Writing – original draft, Writing – review & editing. GZ: Writing – original draft, Writing – review & editing. XZ: Writing – original draft, Writing – review & editing. GL: Writing – original draft, Writing – review & editing. YD: Conceptualization, Writing – review & editing. XX: Conceptualization, Writing – review & editing.
